# Tongue squamous cell carcinoma-targeting Au-HN-1 nanosystem for CT imaging and photothermal therapy

**DOI:** 10.1038/s41368-024-00343-7

**Published:** 2025-01-14

**Authors:** Ming Hao, Xingchen Li, Xinxin Zhang, Boqiang Tao, He Shi, Jianing Wu, Yuyang Li, Xiang Li, Shuangji Li, Han Wu, Jingcheng Xiang, Dongxu Wang, Weiwei Liu, Guoqing Wang

**Affiliations:** 1 Department of Oral and Maxillofacial Surgery, Hospital of Stomatology, Jilin University, Changchun, China; 2 Laboratory Animal Center, College of Animal Science, Jilin University, Changchun, China; 3 State Key Laboratory of Supramolecular Structure and Material, College of Chemistry, Jilin University, Changchun, China; 4 State Key Laboratory for Diagnosis and Treatment of Severe Zoonotic Infectious Diseases, Key Laboratory for Zoonosis Research of the Ministry of Education, College of Basic Medical Science, Jilin University, Changchun, China

**Keywords:** Targeted therapies, Nanoparticles

## Abstract

Tongue squamous cell carcinoma (TSCC) is a prevalent malignancy that afflicts the head and neck area and presents a high incidence of metastasis and invasion. Accurate diagnosis and effective treatment are essential for enhancing the quality of life and the survival rates of TSCC patients. The current treatment modalities for TSCC frequently suffer from a lack of specificity and efficacy. Nanoparticles with diagnostic and photothermal therapeutic properties may offer a new approach for the targeted therapy of TSCC. However, inadequate accumulation of photosensitizers at the tumor site diminishes the efficacy of photothermal therapy (PTT). This study modified gold nanodots (AuNDs) with the TSCC-targeting peptide HN-1 to improve the selectivity and therapeutic effects of PTT. The Au-HN-1 nanosystem effectively targeted the TSCC cells and was rapidly delivered to the tumor tissues compared to the AuNDs. The enhanced accumulation of photosensitizing agents at tumor sites achieved significant PTT effects in a mouse model of TSCC. Moreover, owing to its stable long-term fluorescence and high X-ray attenuation coefficient, the Au-HN-1 nanosystem can be used for fluorescence and computed tomography imaging of TSCC, rendering it useful for early tumor detection and accurate delineation of surgical margins. In conclusion, Au-HN-1 represents a promising nanomedicine for imaging-based diagnosis and targeted PTT of TSCC.

## Introduction

The global annual incidence of oral cancer exceeds 300 000 cases, and the 5-year survival rate ranges from 50% to 60%, resulting in approximately 145 400 deaths per year.^1–4^ Tongue cancer accounts for nearly 40% of these cases,^5^ and its most prevalent form is tongue squamous cell carcinoma (TSCC).^6^ Studies show that the prevalence of TSCC has been increasing in recent years.^7,8^ Furthermore, TSCC exhibits a high incidence of regional lymph node metastasis and local recurrence, which contribute to its aggressive and highly malignant nature.^9,10^ The current primary treatment approach for TSCC is surgical intervention, often complemented by adjuvant therapies such as radiotherapy and chemotherapy.^11^ However, surgical removal of TSCC tissues can significantly impair the essential functions of the tongue, such as respiration, articulation, and mastication.^12^ The surgical resection of TSCC necessitates a precise assessment of the tumor margin; excessive resection would impede functional tongue reconstruction, while inadequate resection may lead to recurrence.^13,14^ Therefore, it is crucial to develop minimally invasive and targeted therapies for TSCC in order to enhance survival rates and overall well-being.

Gold (Au) is extensively used in nanomedicine due to its biocompatibility and low cytotoxicity.^15,16^ In addition, owing to its remarkable ability to convert light into heat, there is considerable interest in developing Au-based nanoplatforms for photothermal therapy (PTT) against various malignancies, including head and neck cancer and prostate cancer.^17,18^ Au nanomaterials have also been utilized as contrast agents in computed tomography (CT) imaging due to enhanced X-ray absorption, thereby exhibiting potential for cancer diagnosis.^19,20^ Furthermore, the size of Au nanomaterials can be tailored to facilitate surface modification, which makes them ideal carriers for cancer theranostics.^21–23^

Although several studies have reported the clinical application of Au nanomaterials in cancer treatment,^24,25^ these nanoparticles lack active targeting ability and rely on passive accumulation in the tumor tissues.^26^ Therefore, development of Au nanomaterials with precise targeting abilities may offer a new treatment approach for TSCC. The tumor-specific internalizing peptide HN-1 is selectively taken up by TSCC cells,^27,28^ and shows limited uptake in normal cells.^29^ In addition, HN-1 can penetrate the entire tumor mass, as demonstrated for head and neck squamous cell carcinoma (NSCC), rather than being restricted to the tumor periphery.^30^ Moreover, HN-1 facilitates nanoparticle delivery, and was able to enhance the therapeutic efficacy of doxorubicin (DOX) against oral squamous cell carcinoma (OSCC) by actively targeting the tumor cells.^31^ Thus, modification of Au nanomaterials with HN-1 may improve their ability to target TSCC cells and enhance therapeutic potential.

**Scheme 1:**
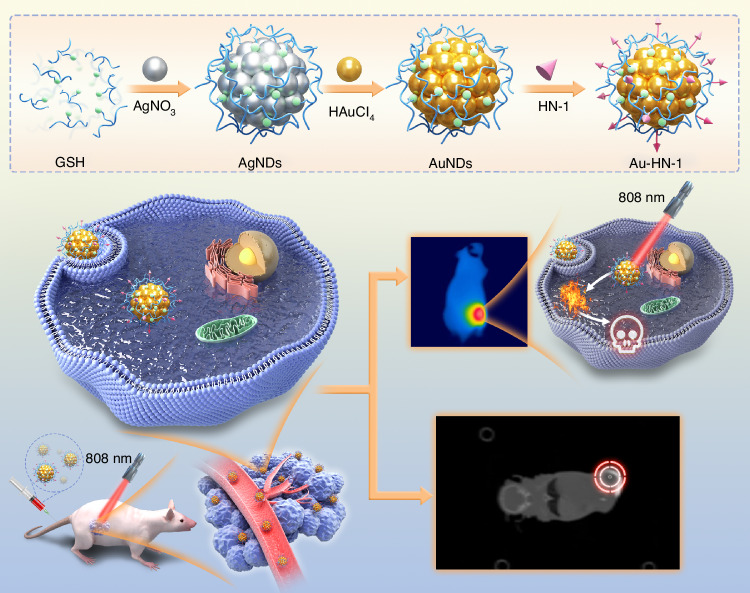
Illustration of the synthesis of Au-HN-1 and its application in tumor site imaging and PTT therapy

This study aimed to design and synthesize an Au-HN-1 nanosystem for TSCC diagnosis and PTT (Scheme 1). To this end, this study developed a formulation combining Au nanodots with the HN-1 targeting peptide, resulting in enhanced CT imaging and PTT effects for high-precision treatment of TSCC. Au-HN-1 demonstrated high targeting specificity for TSCC, and allowed precise visual localization of the tumor through high-contrast CT imaging. Furthermore, Au-HN-1 effectively suppressed the growth of TSCC cells and triggered apoptosis in vitro, and achieved targeted photothermal ablation of the TSCC tumor tissue in vivo in response to near-infrared (NIR) irradiation.

## Results and discussion

### Synthesis and characterization of Au-HN-1

**Fig. 1:**
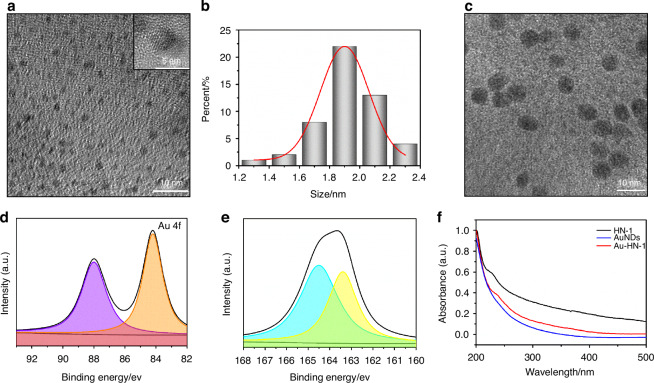
Morphology and structure of the samples. **a** TEM image of the prepared AuNDs, with an inset showing a close-up of the crystal structure of individual AuNDs. **b** Histogram depicting the relative size distribution of AuNDs, based on measurements of 100 nanoclusters. **c** TEM image of the synthesized Au-HN-1. High-resolution XPS spectra of Au 4f (**d**) and S 2p (**e**) of Au-HN-1. **f** UV–Vis absorption spectra of AuNDs, HN-1, and Au-HN-1

The synthesis and application of the Au-HN-1 nanosystem have been described in Scheme 1. Initially, silver nanodots (AgNDs) were fabricated using glutathione (GSH) as the ligand and reducing agent. Subsequently, the AgNDs were etched with Au through an electrochemical exchange reaction owing to the higher redox potential of Ag^+^/Ag (0.80 V versus SHE),^32^ and the resulting AuNDs were then linked with HN-1 through amidation to obtain Au-HN-1. The morphology, size, distribution, and microstructure of the AuNDs and Au-HN-1 were characterized by TEM and nanoparticle measurements (Fig. 1). The AuNDs exhibited a spherical morphology with an average diameter of 1.9 nm and a lattice spacing of 2.35 Å (Fig. 1a, inset), which is consistent with the (111) plane of face-centered cubic Au. The particle size distribution was relatively uniform. Au-HN-1 retained the spherical shape but was considerably larger (6.7 nm), indicating successful conjugation of HN-1 to the AuNDs (Fig. 1b).

The chemical composition of Au-HN-1 was determined by XPS. As shown in Fig. S1, the XPS spectrum of Au-HN-1 included the characteristic peaks of O 1s, S 2p, C 1s, and Au 4f. The high-resolution XPS spectra of Au 4f displayed peaks at 88.0 and 84.2 eV, which corresponded to Au(0) and Au(I) respectively (Fig. 1d). The presence of Au(0) facilitates the nucleation and growth of the nanoparticles, providing a framework for their formation. Furthermore, Au(I) can react with thiols to form stable Au-S bonds, which not only enhance the stability of the nanoparticles but also bestow fluorescence properties, rendering them appropriate for bioimaging and sensing.^33^ These results indicated that the electrochemical exchange reaction effectively reduced Au(III) to lower oxidation states. The binding energies of the S 2p peaks at 163.4 and 164.5 eV were associated with the formed metal–thiol compounds (Fig. 1e).

**Fig. 2:**
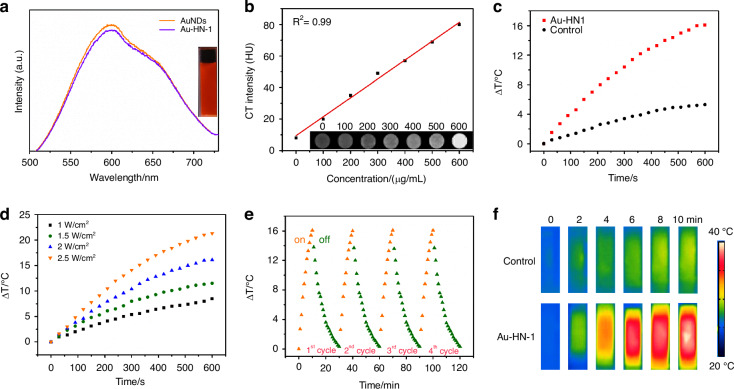
**a** Emission spectra of AuNDs and Au-HN-1 excited at 378 nm. The inset shows a photograph of Au-HN-1 under UV (right) light (*λ* = 365 nm). **b** CT values (HU) of various concentrations of Au-HN-1. The inset shows CT images of Au-HN-1 at different mass concentrations (0, 100, 200, 300, 400, 500, 600 μg /mL). **c** Heating curves of Au-HN-1 compared to PBS (control) under 808 nm laser irradiation (2 W /cm^2^). **d** Temperature changes of Au-HN-1 under various laser power densities for 10 min. **e** Photothermal stability of Au-HN-1. **f** Infrared thermal images of PBS and Au-HN-1 under 808 nm laser irradiation (2 W /cm^2^) for 10 min

The optical characteristics of Au-HN-1 were additionally analyzed by UV-visible spectroscopy. HN-1 exhibited a characteristic peak at 230 nm, while the AuNDs did not display any notable peaks across the measured spectrum (Fig. 1f). Au-HN-1 presented a significant characteristic peak at the relevant wavelength, indicating successful linkage of HN-1. The zeta potential analysis of AuNDs revealed a negative charge of -15.5 mV (Fig. S2), likely due to the COOH groups in GSH, which created a protective shell that inhibited the excessive growth of these NDs. HN-1 and Au-HN-1 exhibited zeta potentials of -4.5 mV and -25 mV respectively. The FTIR spectrum of Au-NH-1 displayed a characteristic vibration peak of -CONH at 1 690 cm^-1^ (Fig. S3), which confirmed the successful conjugation of AuNDs and HN-1. Furthermore, the fluorescence spectrum of Au-HN-1 showed an emission peak at 600 nm (Fig. 2a), indicating that the AuNDs retained their fluorescence properties after conjugation with HN-1. The bright red fluorescence emitted by the particles under UV light (365 nm) (Fig. 2a, inset) is conducive to their application as fluorescent probes for in vivo imaging. To further evaluate the applicability of Au-HN-1 in biomedical imaging, particularly its fluorescence stability, we evaluated its key properties in simulated in vivo environments. To this end, the AuNDs were dissolved in a 20% aqueous solution of Wistar rat serum, PBS, and saline solution. The fluorescence intensity of the AuNDs was unaffected after 24 h (Fig. S4), and only a slight reduction was observed upon UV irradiation, indicating an absence of photobleaching. These results confirm the biocompatibility, stability, and adaptability of the nanosystem, demonstrating its potential as a fluorescence tracer for long-term in vivo biomedical imaging. Au has a high X-ray attenuation coefficient, which renders Au-HN-1 as an attractive contrast agent for enhanced CT imaging.^34–37^ Indeed, the CT intensity of Au-HN-1 increased in a concentration-dependent manner, showing a positive linear relationship (R^2^ = 0.99) (Fig. 2b). Furthermore, the gradual change in grayscale from black to white was indicative of an increase in the X-ray absorption coefficient. These results show that Au-HN-1 is a promising dual-mode imaging probe for both fluorescence lymphography (FL imaging) and CT, positioning it as a valuable asset for early tumor imaging, detection, and therapeutic guidance.

PTT is a promising approach for cancer treatment on account of its non-invasive nature, high spatiotemporal resolution, and minimal toxicity.^38,39^ During PTT, a photothermal agent converts light energy to heat when irradiated with specific light wavelengths. The localized hyperthermia causes protein denaturation, DNA damage, and disruption of the cell membrane, ultimately leading to the selective ablation of tumor tissue. AuNDs are ideal candidates for PTT due to their excellent photothermal properties. The UV-Vis spectrum of Au-HN-1 indicated significant absorption within the NIR range, which is suggestive of photothermal conversion capacity (Fig. S5). As shown in Fig. 2c, the temperature of the Au-HN-1 solution increased rapidly and significantly compared to PBS following exposure to laser irradiation (808 nm, 2 W/cm²) for 10 minutes. NIR irradiation at power densities of 1, 1.5, 2, and 2.5 W/cm^2^ for a duration of 10 min increased the temperature of Au-HN-1 by 8.5 °C, 11.5 °C, 16 °C and 21.5 °C respectively (Fig. 2d). Furthermore, the maximum temperature change in the Au-HN-1 solution was minimal after four successive on/off laser cycles (Fig. 2e), suggesting that the Au-HN-1 nanosystem is photothermally stable and can be used repeatedly. The temperature difference between Au-HN-1 and PBS was also visually apparent in the infrared thermal images (Fig. 2f).

### Cell targeting and anti-tumor effects of Au-HN-1

**Fig. 3:**
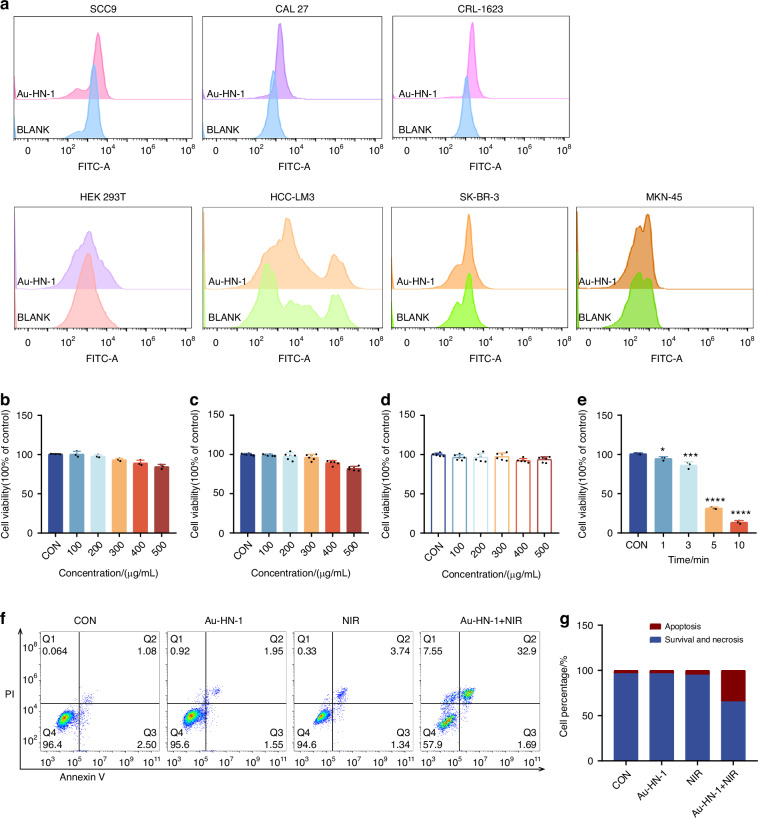
Evaluation of targeting and tumor cell killing effect in vitro. **a** Cellular internalization of Au-HN-1 evaluated by flow cytometry. Cytotoxic effects of **b** AuNDs and **c** Au-HN-1 on SCC9 cells. **d** Cytotoxic effects of Au-HN-1 on HEK 293T. **e** PTT effects of Au-HN-1. **f** Apoptosis analysis. **g** Statistical analysis of apoptosis

**Fig. 4:**
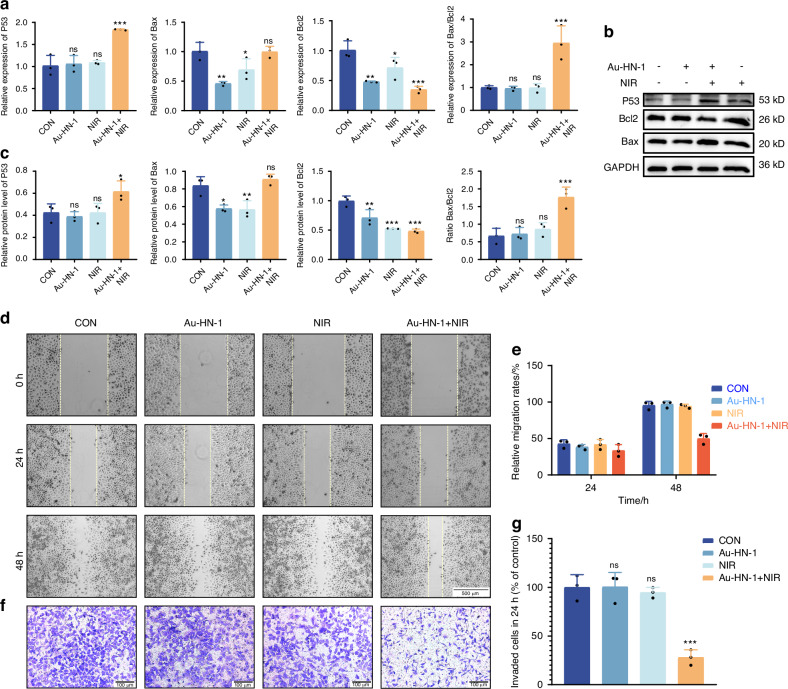
Effects of Au-HN-1 on apoptosis, migration, and invasion of cells. **a** Levels of mRNA expression for P53, Bax, and Bcl2 quantified by qPCR. **b**, **c** Protein expression levels of P53, Bax, and Bcl2 assessed via Western blotting. **d** Evaluation of cell migration and **e** corresponding data analysis. **f** Evaluation of cell invasion and **g** corresponding data analysis

In vitro characterization has confirmed the photothermal efficacy of Au-HN-1, suggesting a favorable therapeutic impact for the treatment of TSCC. Subsequent cellular experiments were conducted to validate these findings. The specific targeting ability of Au-HN-1 was confirmed by flow cytometry and live-cell imaging. While multiple TSCC cell lines (SCC9, CAL 27, and CRL-1623) were able to internalize Au-HN-1, the HEK 293T cells and other tumor cell lines (A549, HCC-LM3, SK-BR-3, and MKN-45) exhibited negligible uptake of Au-HN-1 (Figs. 3a and S6). AuNDs and Au-HN-1 did not significantly impact the viability of the tumor cell lines (Fig. 3b, c), and Au-HN-1 did not demonstrate any cytotoxicity against HEK 293T cells (Fig. 3d), thereby underscoring the biosafety of HN-1. To evaluate PTT efficacy of Au-HN-1, the TSCC cells were incubated with 100 μg/mL of the nanoparticles and irradiated with 808 nm laser (2 W /cm^2^). A negative correlation was noted between the duration of irradiation and cell viability, with the latter dropping below 50% after 5 min of irradiation (Fig. 3e). Furthermore, the combination of Au-HN-1 and NIR irradiation significantly increased apoptosis rates (Fig. 3f, g), which was accompanied by the upregulation of intracellular P53, Bax and caspase 3, downregulation of Bcl2, and an increase in Bax/Bcl2 ratio (Figs. 4a–c and S8a–c). Taken together, Au-HN-1 can induce apoptosis in tumor cells through NIR light-mediated photothermal effect.

TSCC is a highly aggressive malignancy with a propensity for distant metastasis and infiltration into adjacent normal tissues.^40,41^ This invasive behavior significantly contributes to the ambiguity of tumor boundaries and low survival rates, impacting the diagnostic delineation of tumor margins and surgical strategies.^42–44^ Cellular assays revealed that Au-HN-1 + NIR significantly inhibited the migration and invasion of TSCC cells in vitro (Fig. 4d–g), demonstrating that Au-HN-1-mediated PTT may effectively reduce the metastasis of TSCC.

### In vivo distribution and therapeutic effects of Au-HN-1

**Fig. 5:**
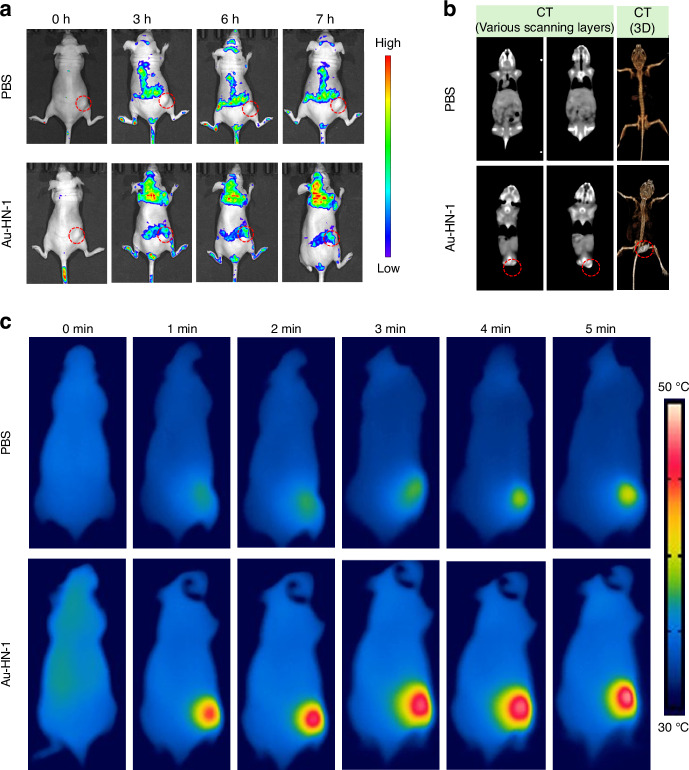
Dual-mode imaging and targeting effect. **a** FL imaging in murine models of TSCC. **b** CT imaging and 3D reconstruction of mice with TSCC after injection of Au-HN-1. **c** Thermal imaging after injection of Au-HN-1 and PBS

**Fig. 6:**
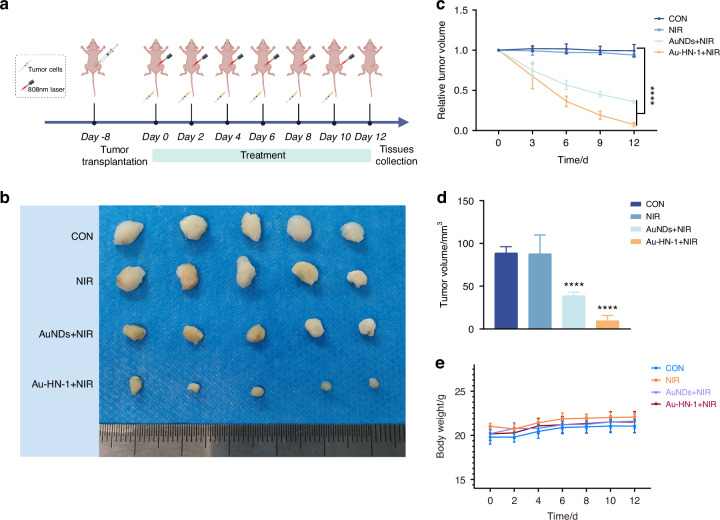
The PTT treatment effect of Au-HN-1. **a** Flow chart illustrating the treatment of murine TSCC. **b** Tumor imaging 12 days post-procedure. **c** 12-day volumetric analysis of the tumor. **d** Analysis of changes in tumor volume over a 12-day period. **e** Analysis of mice’s body weight change over a 12-day period. ****P < 0.000 1

A mouse model of TSCC was developed to assess the therapeutic effects of Au-HN-1 in vivo. The biodistribution of Au-HN-1 was traced by FL imaging. The fluorescence signals of Au-HN-1 were detected at the tumor site within 3 h of inoculation, and intensified at 6 h. Compared to AuNDs, Au-HN-1 exhibited a more precise targeting capability towards the tumor site (Fig. 5a). Furthermore, FL imaging revealed robust accumulation of Au-HN-1 in the tumor tissues, along with metabolism in the liver and kidneys (Fig. S7). Au-HN-1 was also applicable for CT imaging and three-dimensional (3D) reconstruction of tumors (Fig. 5b). This dual functionality can serve as both a supplementary diagnostic aid for surgical intervention, as well as a secondary tool for delineating tumor boundaries. Studies show that PTT can induce direct necrosis of tumor cells at temperatures exceeding 50°C, while lower temperatures (below 50 °C) predominantly induce apoptosis.^45,46^ However, hyperthermia (above 50°C) also causes damage to normal tissues.^47^ Consequently, the ability of HN-1 to specifically target tumor areas can increase the concentration of photosensitizers at those sites and mitigate collateral damage to normal tissues. Consistent with this hypothesis, we detected a significant rise in temperature at the tumor site following Au-HN-1 administration and NIR irradiation (Fig. 5c). The tumor-bearing mice were randomly divided into the subsequent treatment groups: Control (CON), NIR (808 nm, 2 W /cm^2^, 5 min), AuNDs + NIR (808 nm, 2 W /cm^2^, 5 min), and Au-HN-1 + NIR (808 nm, 2 W /cm^2^, 5 min). After 12 days of treatment, the mice were weighed, and the tumor growth was recorded. The combination of Au-HN-1 and NIR radiation significantly reduced tumor volume (Fig. 6a–d) without inducing any appreciable weight loss throughout the treatment period (Fig. 6e), indicating lack of systemic toxicity in vivo.

**Fig. 7:**
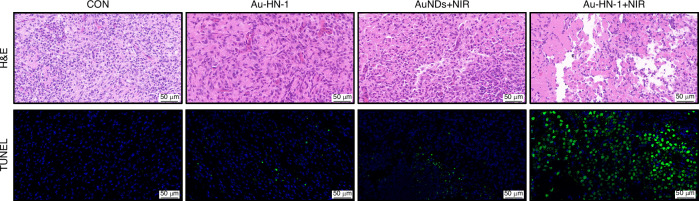
H&E staining and TUNEL staining of tumor tissue

Post-treatment biopsies were conducted for the tumor tissues and the major organs, including heart, liver, spleen, lungs, and kidneys for histological assessment and apoptosis (TUNEL) staining. Au-HN-1 + NIR therapy effectively ablated tumor cells by inducing apoptosis (Fig. 7) without causing any damage to healthy tissues (Fig. S9). In summary, Au-HN-1 achieved targeted elimination of TSCC via NIR-driven PTT, and demonstrated favorable biosafety.

## Discussion and conclusion

Although PTT represents a promising treatment strategy for TSCC, the aggregation of Au nanomaterials at the tumor site primarily relies on passive targeting through the enhanced permeability and retention (EPR) effect.^48^ The development of active targeting strategies for Au would be critical in enhancing their selective accumulation in tumor sites and minimizing off-target effects on healthy tissues. For instance, conjugation of tumor-targeting ligands to Au can facilitate their entry into the tumor cells through the interaction with surface receptors.^49,50^ Peptides in particular are ideal ligands for targeting tumor cells owing to their high stability, affinity, and low immunogenicity.^51^ Previous report indicated that the 12-mer peptide HN-1 (TSPLNIHNGQKL) exhibits specificity towards OSCC cells.^27^ The cellular internalization mechanism of HN-1 has been primarily investigated in terms of ligand-receptor binding and non-receptor binding.^28,52^ The targeting of HN-1 was investigated in the study, revealing its specific uptake by TSCC (SCC9, CAL 27, CRL-1623) cells while normal cell lines exhibited no such uptake.

Nanomaterials with imaging ability can be used as a supplementary diagnostic aid for tumor boundary identification, thereby enhancing the early detection rate and the accuracy of surgical resection.^53^ Therefore, several materials have introduced imaging probes into nanoparticles for tracking tumor growth in vivo. For instance, ICG-functionalized nanoparticles achieved NIR II luminescent imaging of oral tumors.^54^ The optical characteristics of AuNPs can be harnessed for CT imaging of tumors without the need for additional imaging agents.^55^ The present study applied the electrochemical exchange reaction etching method to fabricate AuNDs, which bestowed exceptional optical properties and X-ray attenuation capability. The dual fluorescence and CT imaging ability of AuNPs can be utilized for precise tumor detection and treatment.

Au-HN-1 is a versatile nanosystem for targeted drug delivery and diagnostic imaging. Upon internalization by TSCC cells, Au-HN-1 initiates PTT upon exposure to NIR radiation, resulting in a localized temperature increase at the tumor site and subsequent apoptosis of the tumor cells. Overall, the Au-HN-1 nanosystem is a promising tool for the non-invasive detection and treatment of TSCC.

## Materials and methods

### Materials

Glutathione (GSH), N-hydroxysuccinimide (NHS), tetrachloroauric (III) acid (HAuCl_4_), and 1-ethyl-3-(3-dimethylaminopropyl) carboxylate (EDC) were purchased from Sigma-Aldrich. Isopropyl alcohol, acetone, silver nitrate (AgNO_3_), and hydrazine hydrate (N_2_H_4_·H_2_O) were purchased from Sinopharm Chemical Reagent Co., LTD (Beijing, China).

### Synthesis of AgNDs

To synthesize AgNDs, 0.082 6 g AgNO_3_ and 0.163 5 g GSH were added to 15 mL deionized water, and the solution was stirred for 30 min till a cloudy gel was obtained. The pH of the Ag-GSH gel was adjusted to 6-7 using 1 mol/L NaOH, and 1 mL N_2_H_4_·H_2_O was added. After stirring in the dark for 48 h, excess isopropyl alcohol was added to the mixture, followed by centrifugation, after which the supernatant was removed. The AgNDs were finally obtained by redissolving the precipitate in 9 mL deionized water.

### Synthesis of AuNDs

To synthesize Au NDs, 250 μL of a 50 mol/L HAuCl_4_ solution and 500 μL AgNDs were sequentially added to 12 mL deionized water. The pH of the solution was modified to a range of 7 to 8 by adding 1 mol/L NaOH. After heating at 80 °C for 4 h, the solution was centrifuged at 8 700 r/min for 13 minutes, and the supernatant was mixed with isopropanol. The precipitate was collected by centrifuging at 6 500  r/min for 10 min, and then redispersed in 8 mL deionized water to obtain the AuNDs solution.

### Synthesis of Au-HN-1

To synthesize Au-HN-1, 4 mg NHS and 12 mg EDC were added to 2 mL AuNDs solution and stirred for 20 min. Following addition of 100 μL HN-1 (5 mg/mL), the mixture was left undisturbed for 12 h and then dialyzed in a dialysis bag with a molecular weight cutoff of 3 500 Da for another12 h to obtain the Au-HN-1 solution.

### Characterization of Au-HN-1

Transmission electron microscopy (TEM) imaging was conducted using the JEOL TECNAI F20 instrument set to an operating voltage of 200 kV. The UV-Visible (UV-Vis) absorption spectrum was recorded using Lambda 800 UV-Vis spectrophotometer. Fourier transform infrared (FTIR) spectroscopy was performed over the range of 500 to 4 000 cm^−1^ using Nicolet Avatar 360 FTIR spectrophotometer. Laser scanning confocal microscopy was performed with the Olympus Fluoview FV1000 confocal microscope. X-ray photoelectron spectra (XPS) analysis was performed using the VG ESCALAB MKII spectrometer, with excitation provided by Mg Kα radiation at 1253.6 eV. Fluorescence properties were assessed with Shimadzu RF-5301 PC fluorescence spectrometer. Infrared thermal imaging was performed with FLIR T420 infrared thermal imaging camera.

### Cell culture

Human cell lines of TSCC (SCC9, CAL 27, CRL-1623), non-small cell lung cancer (A549), hepatocarcinoma (HCC-LM3), gastric cancer (MKN-45) and breast cancer (SK-BR-3), and the human embryonic kidney cell line (HEK 293 T) were gained from ATCC and Shanghai Zhong Qiao Xin Zhou Biotechnology. The cells were cultured in DMEM/F12(Gibco, Grand Island, NY,USA), Dulbecco’s modified Eagle medium (DMEM; Gibco, Grand Island, NY,USA)), or RPMI 1640 (Gibco, Grand Island, NY,USA) containing 10% fetal bovine serum (Gibco, Grand Island, NY,USA) and 1% penicillin-streptomycin (Gibco, Grand Island, NY,USA) at 37 °C and 5% CO_2_.

### Cell Counting Kit-8 assay

Cell viability was assessed by the CCK-8 assay (Meilune, Dalian, China). Briefly, SCC9 (3×10^3^) and HEK 293 T cells (5×10^3^) were seeded into 96-well plates and allowed to culture for 24 h, and then incubated with varying concentrations of Au-HN-1 (0, 100, 200, 300, 400 and 500 mg/mL) for 4 h. The cells were irradiated with NIR light at the wavelength of 808 nm for varying durations (60 s, 180 s, 300 s, and 600 s; *n* = 3 per group), and a non-irradiated control was also included (*n* = 3). Complete medium containing 10% CCK-8 solution was added to each well, and the cells were then incubated for another hour. The absorbance was measured at 450 nm, and the proliferation rates were calculated for the different drug concentrations.

### Apoptosis assay

SCC9 cells were treated with Au-HN-1 (100 μg/mL) or the vehicle and then exposed to 808 nm NIR light at the intensity of 2 W/cm^2^ for 5 min; respective non-irradiated controls were also included. After a 24 h period of cell treatment, the cells were harvested from the cell culture dishes. After rinsing twice with PBS, the cells were stained using propidium iodide and Annexin V-FITC as per the kit instructions (PI; Beyotime, China). The apoptotic cells were detected by flow cytometry (BD Biosciences, USA). Each experiment was conducted in three replicates.

### Cell migration assay

The wound-healing assay was utilized to evaluate cell migration. SCC9 cells were seeded in 6-well plates at the density of 1 × 10^5^ cells per well and incubated for 24 h. Following treatment with Au-HN-1 (100 μg/mL) and 808 nm NIR irradiation (2 W/cm^2^ 5 min) as described above, the monolayer was scratched to create a “wound” and cultured without FBS. Images of the wound area were captured at 0, 24, and 48 hours to evaluate cell migration. The captured images are processed using ImageJ software to quantify the extent of wound closure. Each experiment was conducted in three replicates.

### Cell invasion assay

Cell invasion assays were performed using SCC9 cells (1 × 10^4^). The cells were treated with Au-HN-1 (100 μg/mL), 808 nm NIR light (2 W/cm^2^, 5 min), or Au-HN-1 (100 μg/mL) + 808 nm NIR light (2 W/cm^2^, 5 min) for 24 h. After treatment, these cells were routinely digested using pancreatic enzymes, then counted separately using cell counters, and subsequently resuscitated utilizing serum-free medium. The cells were subsequently seeded into the wells. The wells were pre-treated with Matrigel matrix (10 μL, BD Biosciences, USA) and incubated overnight in the presence of medium. The lower cell compartment was immersed in a medium containing serum. The cells seeded into the wells were incubated for 24 hours. Then the invaded cells were stained using 0.1% crystal violet dye (Solarbio, China). The stained cells are rinsed with water to eliminate any residual dye. The cells that had been stained were subsequently subjected to analysis using the ImageJ software, developed by the National Institutes of Health (NIH) in the United States. Each experiment was conducted in three replicates.

### Cell targeting

The targeting ability of Au-HN-1 was examined on multiple TSCC cell lines using flow cytometry and laser confocal cell imaging. Specifically, SCC9, CAL 27, CRL-1623, HEK 293 T, HCC-LM3, SK-BR-3, and MKN-45 cells were plated in 6-well plates, allowed to incubate for 24 h, and subsequently exposed to Au-HN-1 for 6 h. Following three washes with PBS, the cells were re-suspended in buffer and subjected to flow cytometry analysis. A total of 1 × 10^4^ cells were collected for each sample.

The cellular uptake of Au-HN-1 was further tracked by confocal microscopy. The SCC9, A549, and HCC-LM3 cells were seeded into confocal laser plates and treated with Au-HN-1 for 6 h. After washing thrice with PBS, the cells were stained with DAPI for 10 min, washed three times with PBS to eliminate any residual dye, and imaged under a confocal laser scanning microscope (Carl Zeiss AG, Germany).

### Quantitative real-time PCR

The SCC9 cells were subjected to a 24-hour treatment, followed by extraction of RNA for the purpose of gene expression analysis. Total RNA was extracted using the TRIzol reagent (Invitrogen) and reverse transcribed to cDNA using an RNA reverse transcription kit (Genesand Biotech Co., Ltd., China). Quantitative PCR (qPCR) was performed using the GS AntiQ SYBR Green Fast Mix Kit (Genesand Biotech Co., Ltd., China). The cycling conditions were as follows: 95 °C for 15 min followed by 40 cycles of 95 °C for 15 s, 60 °C for 30 s, and 70 °C for 20 s. The relative gene expression levels were calculated by the 2^–ΔΔCt^ method. Each assay was performed in triplicate. The primer sequences are listed in Table SI.

### Western blotting

The protein fraction was extracted from the suitably treated SCC9 cells using a protein extraction buffer (Beyotime, China) and quantified with the BCA protein assay kit (Tiangen, China). The extracted protein should be supplemented with loading buffer and subjected to boiling for 10 minutes in order to achieve denaturation of the protein. The proteins were separated by SDS-PAGE using 10% gel and then transferred onto PVDF membranes. After blocking with 5% non-fat milk powder, the membranes were washed with TBST (0.1% Tween-20) and incubated overnight with anti-p53 (Cell Signaling Technology, 2524, USA), anti-Bcl2 (Cell Signaling Technology, 15071, USA), anti-Bax (Cell Signaling Technology, 2772, USA), anti-caspase 3 (ZEN-BIOSCIENCE, R23315, China) and anti-GAPDH (Affinity, AF7021, USA) antibodies at 4 °C. Subsequently, the membranes were incubated with HRP-conjugated affiniPure goat IgG antibodies (BOSTER, China) for 2 h. The protein bands were developed using the ECL Super Signal kit (Pierce, Thermo Fisher Scientific, USA). The gray values of the bands were analyzed for protein level analysis using ImageJ software. Each experiment was conducted in three replicates.

### In vivo targeting

Female nude mice (6-8 weeks) were acquired from the Laboratory Animal Center of Jilin University (Approval number: SY202310026). All animal procedures were conducted in accordance with the guidelines of the National Regulation of China for Care and Use of Laboratory Animals. The mice were kept in a specific pathogen-free environment at 24 °C, with a relative humidity of 50%-60%, and maintained on a 12 h light/dark cycle with free access to standard rodent diet and fresh water. Throughout the experiment, all mice remained healthy and uninfected. All surgical procedures were conducted under sterile conditions. The animals that exhibited abnormal eating patterns, rapid weight loss, or cachexia were euthanized through CO_2_ inhalation.

The mice were inoculated with SCC9 cells (8×10^6^ cells per animal) into their right flank to establish a tumor model. Tumor-bearing mice received intravenous injections of Au-HN-1 (100 μL of 1 mg/mL solution) or AuNDs (100 μL of 1 mg/mL solution), and imaged at specified intervals using the IVIS Lumina LT imaging system. The mice were euthanized 24 h after administering the drugs, and the hearts, livers, spleens, lungs, kidneys, and tumors were harvested and imaged.

### Evaluation of in vivo anti-tumor efficacy

Female nude mice (*N* = 20, 6–8 weeks old) were injected with SCC9 cells (8×10^6^ cells per animal) into their right flank to establish TSCC xenografts. The tumor-bearing mice were divided into the following groups: Control (CON), NIR (808 nm, 2 W/cm^2^, 5 min), AuNDs + NIR (808 nm, 2 W/cm^2^, 5 min), and Au-HN-1 + NIR (808 nm, 2 W/cm^2^, 5 min). The mice were euthanized after 12 days of treatment, and the tumors and vital organs were collected for histological analysis. The tumor dimensions were measured, and the volume (V) was calculated as a × b^2^/2, where a is the longer diameter and b is the shorter diameter. To evaluate the biosafety of the formulations, the mice were regularly weighed, and the major organs were histologically examined for any signs of toxicity.

### Statistical analysis

Each experiment was conducted three times. Statistical evaluations were carried out using GraphPad Prism software (version 9). The groups were compared by Students’ t-test, one-way analysis of variance (ANOVA) followed by Dunnett’s multiple comparisons test, and two-way ANOVA followed by Tukey’s multiple comparisons test. A *P*-value of less than 0.05 was deemed to be statistically significant.

## Supplementary information

Supporting Information

Supplementary Table SI
