# Supplementary material for: Tongue squamous cell carcinoma-targeting Au-HN-1 nanosystem for CT imaging and photothermal therapy
Source: Int J Oral Sci. 2025 Jan 14;17:9. doi: 10.1038/s41368-024-00343-7 (PMC11729884; doi:10.1038/s41368-024-00343-7)
Supplement: Supplementary file 1 — Supporting Information [file 41368_2024_343_MOESM1_ESM.docx]

**Supporting Information**





**Figure S1.** XPS survey spectrum of Au-HN-1.





**Figure S2.** Zeta potential.


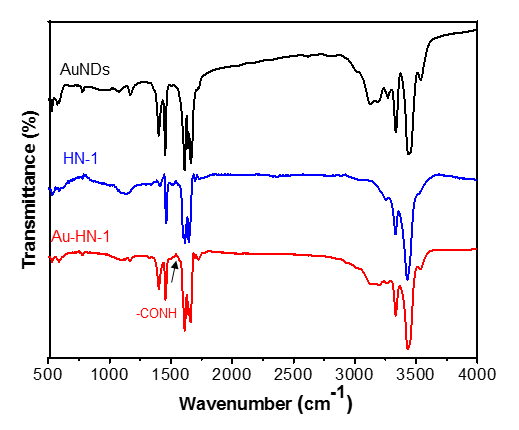


**Figure S3.** FTIR spectra.





**Figure S4.** Fluorescence intensities of AuNDs dispersed in PBS, 20% serum, water and exposed to UV irradiation changed at different time, respectively.





**Figure S5.** UV−vis absorption spectra of Au-HN-1.


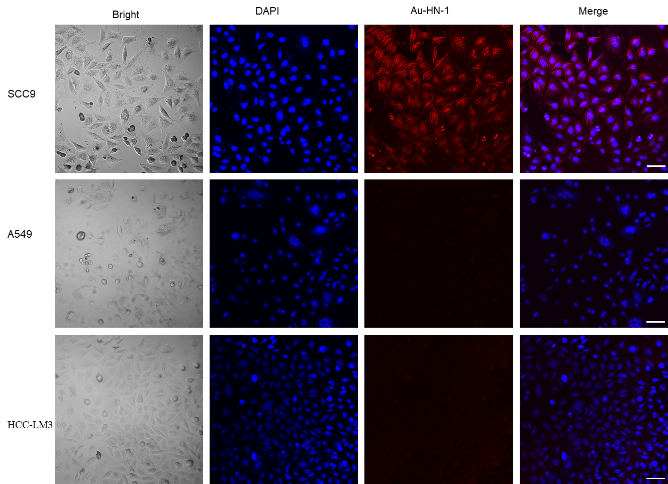


**Figure S6.** Laser confocal image of Au-HN-1 targeting. Scale bar：50μm


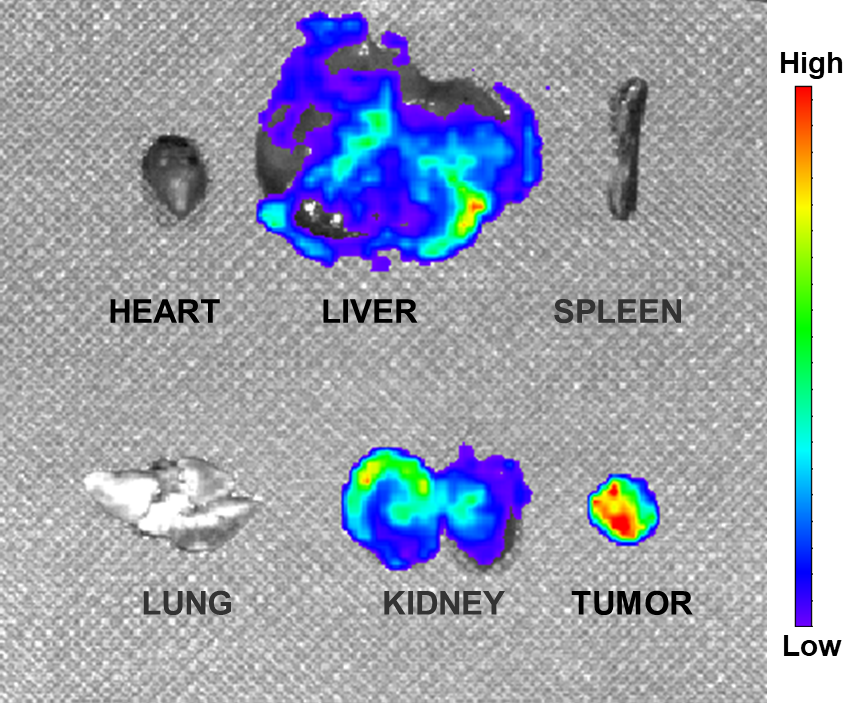


**Figure S7.** FL imaging of mouse tumors and vital organs.


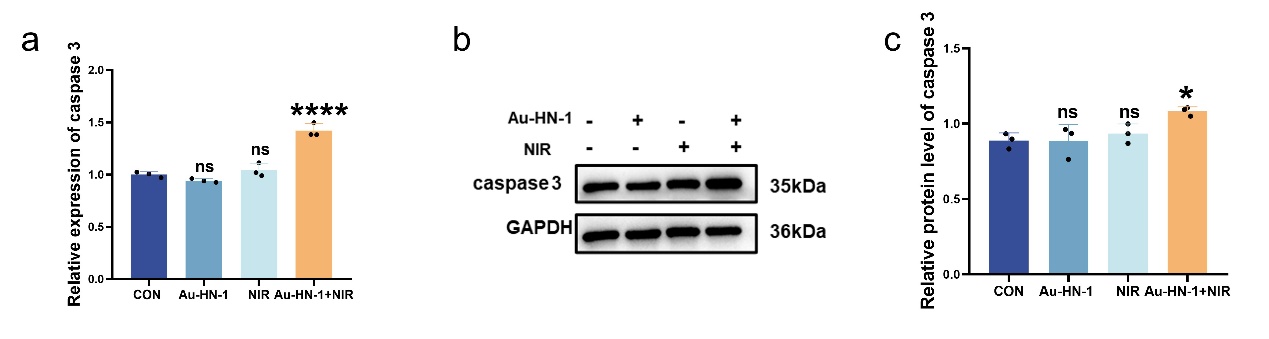
**Figure S8.** The expression level of caspase 3. (a) Relative expression of *caspase 3*. (b) Protein expression level of caspase 3. (c) Analysis of caspase 3 protein level.


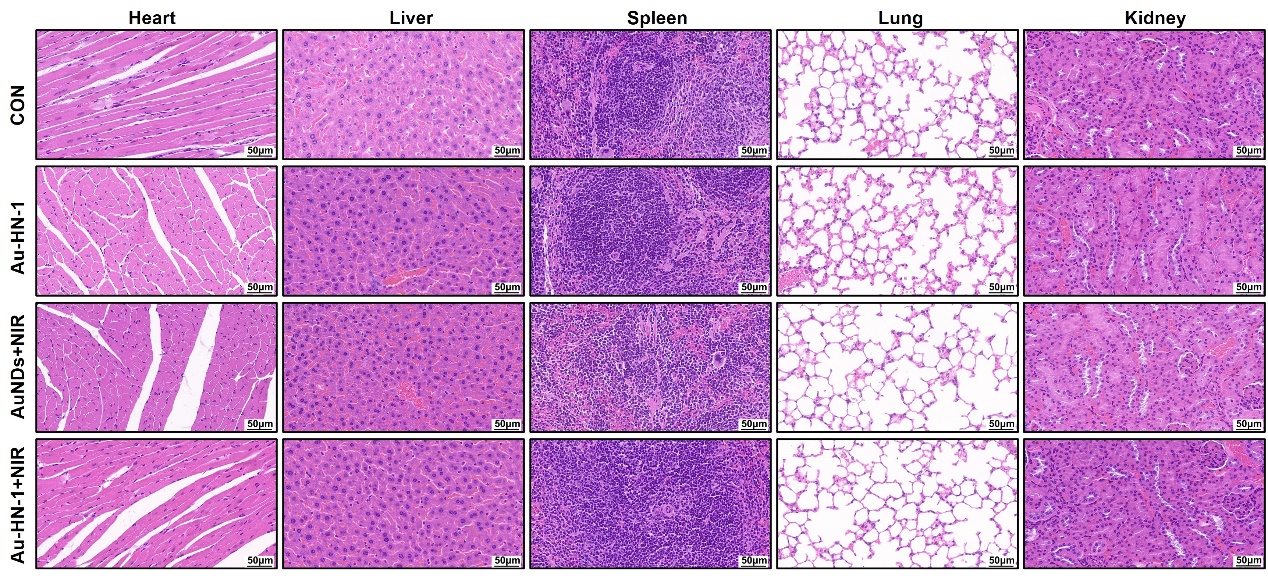


**Figure S9.** H&E staining of major organs post-treatment.
