# Supplementary material for: Tongue squamous cell carcinoma-targeting Au-HN-1 nanosystem for CT imaging and photothermal therapy
Source: Int J Oral Sci. 2025 Jan 14;17:9. doi: 10.1038/s41368-024-00343-7 (PMC11729884; doi:10.1038/s41368-024-00343-7)
Supplement: Supplementary file 2 — Supplementary Table SI [file 41368_2024_343_MOESM2_ESM.docx]

Table SI. qPCR primer sequences

| Genes |  | Primer sequences (5’→3’) |
| --- | --- | --- |
| *h-GAPDH* | F： | TGGTATCGTGGAAGGACTCA |
|  | R： | GGGCCATCGACAGTCTTC |
| *h-Caspase 3* | F： | CATGGAAGCGAATCAATGGACT |
|  | R： | CTGTACCAGACCGAGATGTCA |
| *h-Bax* | F： | CCCGAGAGGTCTTTTTCCGAG |
|  | R： | CCAGCCCATGATGGTTCTGAT |
| *h-Bcl2* | F： | GGTGGGGTCATGTGTGTGG |
|  | R： | CGGTTCAGGTACTCAGTCATCC |
| *h-* *TP53* | F： | CAGCACATGACGGAGGTTGT |
|  | R： | TCATCCAAATACTCCACACGC |
